# Correlations between children’s Wushu participation, posture, physical fitness, and visual indicators

**DOI:** 10.3389/fpubh.2026.1950054

**Published:** 2026-10-14

**Authors:** Heming Wang, Ximei Feng, Guohua Li

**Affiliations:** 1 Competitive Sports Center, Taishan College of Science and Technology, Tai'an, Shandong, China; 2 School Hospital, Shanxi University, Taiyuan, Shanxi, China; 3 School of Physical Education, Shanxi University, Taiyuan, Shanxi, China

**Keywords:** children, physical fitness, posture development, retrospective cohort, visual function, Wushu participation history

## Abstract

**Inroduction:**

At present, school‑aged children face three common public‑health concerns: functional postural abnormalities, poor physical fitness, and visual fatigue accompanied by early refractive changes. Targeted exercise programmes that simultaneously mitigate all three health problems remain scarce. This study aimed to examine associations between long‑term participation in a comprehensive standardized Wushu programme containing embedded visual‑relaxation exercises and children’s postural, physical‑fitness, and ocular indicators.

**Methods:**

This retrospective analysis extracted school physical‑and‑vision screening archives of 196 primary‑school students collected between July‑December 2025. According to after‑school activity records, participants were divided into an experimental group (*n* = 98, receiving consistent standardized Wushu training) and a control group (*n* = 98, routine physical education without systematic Wushu practice). Researchers did not provide additional training for participants. Data covering a unified 16‑week observation period were extracted following national standard assessment protocols. Linear mixed‑effects models, partial correlation analysis and forward‑stepwise regression were used, with adjustment for age, gender and daily screen‑reading time.

**Results:**

After 16 weeks of observation, the experimental group demonstrated better body alignment and decreased postural anomalies including uneven shoulder height and trunk tilt (*p* < 0.01). Significantly higher scores for cardiorespiratory endurance, core strength, flexibility and balance were also observed, alongside significant group‑by‑time interactions (*p* < 0.01). Compared with the control group, children participating in the Wushu programme achieved better uncorrected distance visual acuity, ciliary‑muscle accommodation capacity and visual‑fatigue recovery performance (*p* < 0.01). Wushu‑participation frequency and duration were independent positive correlates of improved posture, physical fitness and visual function (*p* < 0.01). Notably, improvements in visual function may partly derive from embedded visual‑relaxation drills rather than Wushu movements alone.

**Discussion:**

Given its retrospective archival design, causal inferences cannot be established. These real‑world correlational findings provide evidence to support school‑sport planning targeting children’s physical and visual health.

## 1 Introduction

Childhood is a critical developmental period for somatic morphology formation, physical foundation construction and visual system maturation. Physical and visual conditions established in childhood exert long-term impacts on individuals’ lifelong physical and psychological wellness. Nowadays, prolonged sedentary study, desk work and frequent electronic device use disrupt children’s natural growth rhythms and trigger widespread physical health risks (1). Functional postural abnormality has become a prevailing developmental issue for school-aged children. The spinal, scapular and trunk musculoskeletal structures of children are immature and highly plastic. Prolonged improper sitting and standing postures, together with insufficient structured physical activity, cause unbalanced core muscle tension and distorted trunk mechanical force lines, further resulting in uneven shoulder height, asymmetric trunk alignment and spinal malalignment (2). Such functional disorders lack organic lesions in the early phase and are easily overlooked by parents and teachers; if left uncorrected over time, they may develop into irreversible skeletal deformities. These deformities create uneven bodily stress distribution, break normal motor compensation patterns and weaken children’s daily mobility and balance capacity (3).

Widespread sedentary habits also reduce children’s daily activity volume, and most daily physical activities are single-mode, lacking integrated coordination and stability training. This leads to weak core muscles, poor body control and insufficient cardiorespiratory reserve, resulting in continuous declines in overall physical fitness (4). Meanwhile, children’s ocular accommodation systems are in a fast-developing, plastic yet fragile stage. Sustained close visual work, fixed viewing angles and inadequate outdoor dynamic visual stimulation trigger persistent accommodative spasm. Unrelieved visual fatigue gradually induces visual decline and refractive shifts (5). Functional postural malalignment, poor physical fitness and impaired visual function have become prominent public health barriers to children’s healthy growth. These three interact to hinder balanced physical development, limit motor ability and disrupt stable posture and vision, bringing long-term adverse effects on children’s physical condition, body shape and self-esteem (6).

Structured physical activity serves an important pathway to relieve children’s postural, fitness and visual problems, and has become a core research focus in child public health. As a traditional integrated Chinese sport, Wushu combines static stances and dynamic routines, unifying bodily movement and mental concentration to coordinate internal and external physical functions (7). Wushu stances and routines require upright trunks and relaxed shoulders, which continuously activate deep lumbar, back and scapular core muscles to balance asymmetric muscle tension and mitigate compensatory postural habits, preventing mild functional postural defects from worsening into organic lesions (8, 9). The integration of static stability training and dynamic rhythmic routines stimulates cardiovascular circulation, improves limb flexibility, core strength and whole-body balance, and makes up for children’s insufficient all-round physical development (10). During Wushu practice, participants follow limb and body movements with their eyes, forming alternating near-far visual stimulation that repeatedly contracts and relaxes the ciliary muscle to boost accommodation flexibility and accelerate visual fatigue relief (11, 12). Such multi-dimensional movement characteristics simultaneously benefit bodily alignment, comprehensive fitness and ocular regulation, making Wushu a valuable optional school sport for children.

Existing studies on Wushu and children’s health rarely conduct comprehensive empirical analyses covering posture, physical fitness and visual function simultaneously, and few explore the associations between long-term Wushu involvement and multi-dimensional physical indicators (8, 13). Based on historical school physical screening archives, this retrospective cohort study systematically analyzes the correlations between long-term Wushu participation records and children’s posture development, physical level and visual function. The findings provide real-world archival reference for assessing long-term physical benefits linked to Wushu participation and optimizing after-school sports arrangements for school-aged children, and offer empirical support for relevant child health promotion initiatives.

## 2 Materials and methods

### 2.1 Research subjects

This study used a retrospective cohort design. All analytical data were extracted from historical physical-examination, vision-screening and after-school-sports registration archives of local public primary schools. The documented 16-week after-school-sports activity cycle spanned August 11 2025 – October 21 2025. Baseline screening was completed in early August 2025 before activity initiation, and post-cycle screening was conducted in late October 2025 immediately upon cycle completion. Data collation was conducted between July and December 2025; researchers did not organize any offline sports activities or newly arranged training for any enrolled children.

A total of 232 children with complete multi-year school health files were initially screened. Twenty-two participants were excluded according to pre-defined exclusion criteria (organic postural disorders = 8, ocular organic lesions or corrective-lens wear = 6, exercise contraindications = 5, inability to complete full follow-up = 3), leaving 210 participants eligible for matching (105 in the Wushu group and 105 in the control group).

One-to-one propensity-score matching was performed with the nearest-neighbor algorithm, sampling without replacement. The caliper threshold was set to 0.2 standard deviations for logit-transformed propensity scores. The propensity-score logistic regression model incorporated ten covariates: age, sex, BMI, baseline comprehensive posture score, baseline average physical-fitness score, baseline binocular uncorrected visual acuity, daily screen-reading duration, daily electronic-device-use duration, daily outdoor light-exposure duration, and sleep duration. We assessed post-matching balance using the standardized mean difference (SMD). An SMD below 0.10 was taken to reflect adequate between-group balance. SMD statistics for each covariate are reported in Table 1.

**Table 1:** Comparison of baseline general data between two groups of children.

Baseline characteristics	Control group (*n* = 98)	Experimental group (*n* = 98)	*t/χ^2^*	*p*	SMD
Age (years)	9.16 ± 1.73	9.08 ± 1.69	0.326	0.745	0.047
Gender [*n* (%)]			0.082	0.774	0.041
Male	52 (53.06)	50 (51.02)			
Female	46 (46.94)	48 (48.98)			
BMI (kg/m^2^)	16.24 ± 2.15	16.18 ± 2.09	0.192	0.848	0.028
Daily reading eye-use duration (h)	3.47 ± 0.62	3.42 ± 0.58	0.571	0.568	0.083
Daily electronic device use duration (h)	1.83 ± 0.41	1.79 ± 0.38	0.705	0.482	0.100
Daily outdoor light exposure duration (h)	1.26 ± 0.34	1.30 ± 0.36	0.794	0.428	0.114
Sleep duration (h)	9.35 ± 0.72	9.41 ± 0.68	0.602	0.548	0.086
Comprehensive posture score (points)	62.74 ± 6.83	63.12 ± 6.75	0.389	0.697	0.056
Average comprehensive fitness score (points)	63.15 ± 6.92	63.06 ± 6.87	0.091	0.927	0.013
Average binocular uncorrected vision	4.81 ± 0.14	4.82 ± 0.13	0.514	0.608	0.074

SMD, standardized mean difference. Most covariates showed SMD < 0.10 after propensity-score matching; SMD values for daily electronic-device-use duration (0.100) and daily outdoor light-exposure duration (0.114) were at or slightly above the 0.10 threshold, although the corresponding *p*-values remained > 0.05.

Fourteen unmatched participants (7 per group) were excluded after matching, and 98 matched pairs (196 participants in total) were finally included in analysis. Participant screening and matching procedures are illustrated in Figure 1.

**Figure 1:**
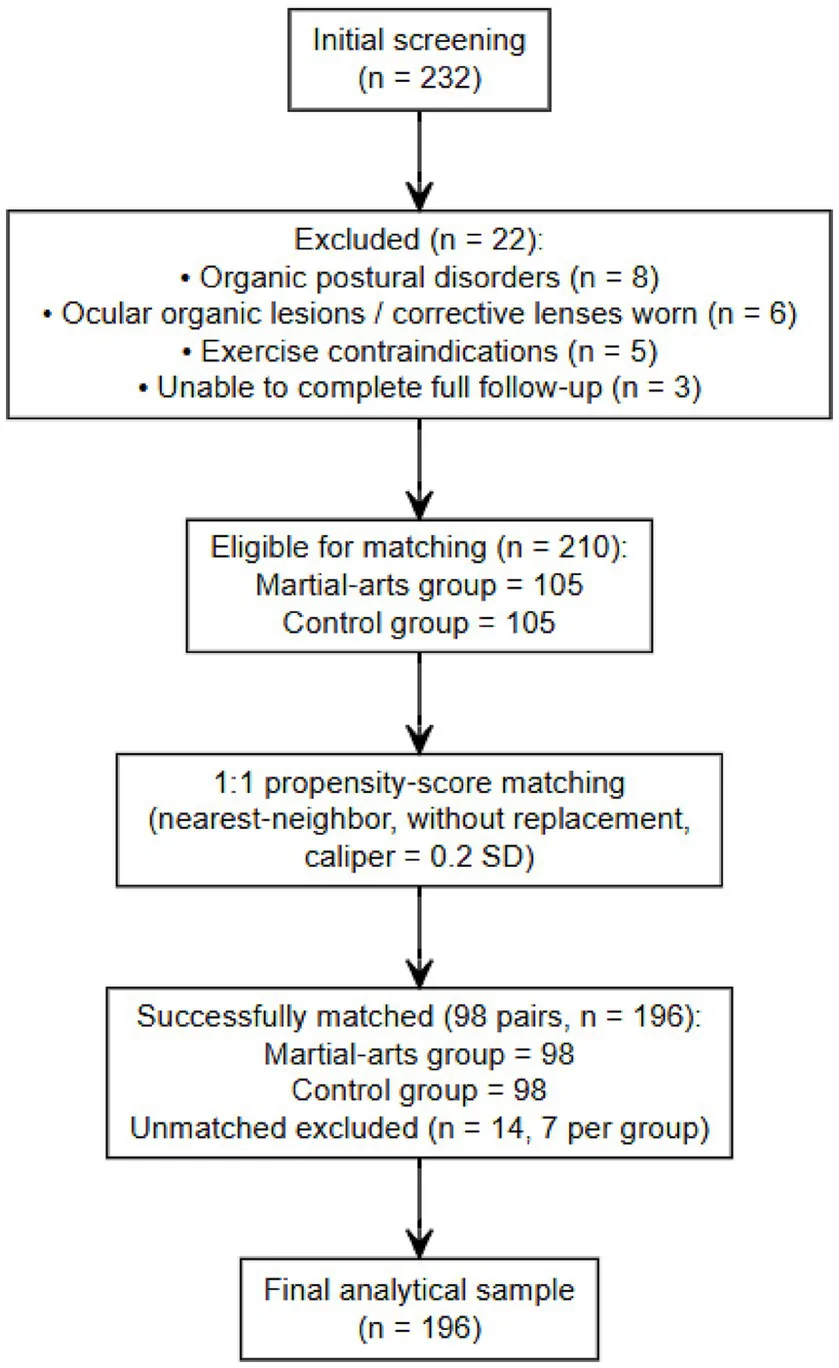
Participant screening and propensity-score matching flow diagram.

All children shared consistent daily schedules, learning environments and living conditions to reduce environmental confounding bias. Baseline indicators covering age, sex, height, weight, BMI, initial posture and fitness levels, baseline vision, daily close-range eye-use and outdoor-activity time exhibited no statistically significant inter-group differences (*p* > 0.05), confirming balanced baseline characteristics between the two groups.

Sample-size calculation took comprehensive posture score as the primary outcome indicator. Statistical power was set to 0.8 with a two-sided α value of 0.05. Referring to relevant published literature, the expected standardized effect size was set at 0.42 (14). Calculations indicated a minimum of 86 participants per group. After accounting for a presumed 15% incomplete-record rate, the required sample size for each group was 98, which was satisfied by the final number of participants in this study.

All physical data analyzed in this paper originated from routine annual school-screening archives. Prior to student admission, schools obtained unified written permission from guardians for data preservation and secondary statistical analysis of screening records. All personally identifiable information was fully desensitized before data extraction to protect participants’ privacy. Researchers had no offline communication with children or their guardians throughout the entire file-sorting process. All guardians received and signed printed informed-consent forms. Guardians reserved the right to withdraw their children’s archival data from this analysis at any time. All participants were fully informed of school physical-screening procedures and data-usage specifications. All original personal files were kept strictly confidential and used solely for statistical analysis in the present study.

### 2.2 Inclusion criteria

1. Participants were school-age children aged between 6 and 12 years old.
2. No records of targeted independent posture correction, physical fitness or vision rehabilitation training were found in subjects’ archives within the six months before the 16-week observation cycle.
3. Participants had no long-term regular high-intensity exercise habits and kept balanced basic movement levels.
4. The school archives contained complete continuous posture, physical fitness and binocular vision screening data covering the corresponding 16-week sports record observation cycle, without missing core indicator records.
5. Participants’ daily electronic device use duration and reading eye use duration matched the average level of school-age children without extreme eye use behaviors.

### 2.3 Exclusion criteria

1. Children with organic posture diseases such as scoliosis and congenital uneven shoulders or asymmetric limbs were excluded from the research.
2. Children diagnosed with asthma, congenital heart disease, myasthenia and other diseases unsuitable for regular physical exercise could not participate.
3. Children with organic eye lesions including astigmatism, amblyopia and glaucoma were ruled out. Participants with pseudomyopia who wore corrective glasses or received professional vision rehabilitation treatment were also excluded.
4. Subjects with discontinuous extracurricular sports records, or those who received other long-term posture correction or vision rehabilitation training within the archived sports observation cycle were excluded.

### 2.4 Definition of group classification standards

Participants were categorized into the experimental group (*n* = 98, holding complete archives of standardized 16-week children’s Wushu activities) and the control group (*n* = 98, with records of only routine general campus sports and no systematic Wushu-participation history).

The exposure variable of this study refers to historical records of standardized children’s Wushu participation archived in school after-school-activity files. The archival activity specification covers a 16-week schedule: three 60-min sessions per week, consisting of static posture stance practice, simplified Wushu routines, and embedded visual-relaxation components. Specifically, the Wushu protocol contained 3 min of alternating far-near gaze training during the warm-up phase and an additional 3 min of distant-gaze relaxation during the cool-down phase. Over the complete intervention cycle, participants accumulated approximately 288 min of structured visual-relaxation drills in total. By contrast, records of participants in the control group only documented general recreational sports including rope skipping, ball games and group calisthenics. Control-group activities did not include any dedicated far-near viewing or distant-gaze relaxation drills.

Accordingly, observed inter-group differences in visual-function outcomes cannot be fully attributed to Wushu motor movements alone; improvements may partly stem from these embedded visual-relaxation exercises. This confounding factor is further elaborated in the Discussion section.

### 2.5 Testing indicators

All posture, physical fitness, and ocular function indicators were measured by trained professional examiners during routine school physical examinations following national unified assessment standards. All raw data were retrospectively extracted from official historical school screening archives. Two waves of standardized screening data were included: baseline data obtained prior to the 16-week observational cycle and post-cycle data collected immediately upon cycle completion. All annual physical examinations adopted consistent examiners, calibrated testing equipment, and unified evaluation criteria. All measurements were implemented by the same professional assessment team with identical testing tools and standardized operational protocols to maximize data consistency and minimize systematic measurement bias. The overall testing system comprised three unified modules: postural indicators, physical fitness indicators, and ocular function indicators.

#### 2.5.1 Posture testing indicators and standards

This research adopted manual standardized visual inspection combined with soft ruler and protractor measurement for children’s posture testing following the General Specification for Student Posture Assessment (15). All tests took place indoors under standard natural light (3). Each participant adjusted to a standard upright static posture before measurement. Subjects stood with feet shoulder-width apart, knees slightly bent without locking, neutral pelvis and naturally relaxed shoulders. Their trunks stayed vertical with eyes looking straight forward. All muscles kept relaxed without compensatory movements such as deliberate chest expansion, abdominal contraction or forward head tilt. Examiners observed and measured participants from front, side and back views. All data were measured three times and averaged to reduce single-measurement errors. The core posture testing indicators and abnormal judgment thresholds followed unified standards. The height difference between bilateral acromions was measured by soft ruler to record vertical distance from each shoulder peak to the ground. A height difference equal to or over 0.5 centimeters was defined as uneven shoulder abnormality. Trunk offset angle was measured with a protractor against the human median sagittal plane. An offset angle equal to or over 3 degrees was judged as trunk offset abnormality. Body posture asymmetry rate focused on symmetric conditions of bilateral acromions, iliac hips, thoracic trunk and standing limbs. Any obvious height, front-back or left–right asymmetry beyond normal physiological deviation was recorded as asymmetric posture abnormality. Thoracic kyphosis angle was measured through spinal surface angle detection. The normal reference range for school-age children stood between 20 degrees and 40 degrees. Thoracic kyphosis over 40 degrees counted as hunchback abnormality while angles below 20 degrees were defined as chest collapse abnormality. Forward head angle referred to the tilt angle between the perpendicular line from the earlobe and shoulder peak. A cervical forward tilt angle over 15 degrees was marked as forward head abnormality. We counted the number and detection rate of each single abnormal posture for the two groups. We also gave a comprehensive score based on overall body regularity, symmetry and uprightness of shoulders, neck, chest, waist and trunk. Two professionally trained examiners completed scoring independently after unified standardized posture assessment training. Inter-rater reliability test was carried out after all sample evaluation. The intraclass correlation coefficient reached 0.912 to prove excellent consistency between two evaluators. The full score of posture regularity was 100 points. Higher scores represented better overall body regularity.

#### 2.5.2 Physical fitness testing indicators and standards

We referenced National Student Physical Health Standard combined with special children’s physical fitness assessment standards to test core dimensions of comprehensive physical fitness (16). Cardiorespiratory endurance was tested through 800-meter running for female children and 1,000-meter running for male children. Examiners recorded completion time and heart rate recovery level to convert cardiorespiratory endurance scores. Core muscle strength was assessed through one-minute sit-up tests with recorded valid completion counts to evaluate abdominal and lumbar core muscle strength. Limb flexibility adopted sit-and-reach tests with precise forward stretch distance recorded to 0.1 centimeters. Balance and coordination ability was measured through single-leg eyes-closed standing tests with recorded maximum standing duration to assess body balance control capacity. Limb coordination was evaluated through one-minute rope skipping tests with valid jump counts recorded to comprehensively assess limb coordination, explosive force and body control ability. All indicators were converted into standard hundred-point scores. Higher scores represented better physical fitness levels.

#### 2.5.3 Eye vision and function testing indicators and standards

All eye-vision and-function tests were performed in accordance with the unified Small and Medium-Sized Student Vision Health Screening Technical Specifications (17). Test rooms were equipped with standard natural light, avoiding intense direct light and reflective interference. Before testing, all children rested quietly in dim light for 10 min to eliminate transient visual fatigue and standardize baseline conditions. All measurements were completed by fixed professional examiners. Each indicator was measured twice, and the average value was adopted to reduce operational measurement error. Core ocular indicators were assessed using standardized testing protocols described below. Before each ocular assessment, examiners confirmed that participants maintained a consistent testing posture and completed the procedures under the same environmental conditions. Any incomplete or invalid measurements were repeated according to the same protocol.

Uncorrected distance visual acuity was measured using a national-standard 5-metre logarithmic visual chart (18). Participants maintained an upright sitting posture, gazing straight ahead without squinting or forward head tilt. Uncorrected visual acuity of the left and right eyes was tested separately and recorded; the mean value of both eyes was taken as individual-level uncorrected visual acuity. The same chart illumination and testing distance were maintained throughout all assessments to ensure comparability between participants.

For the ocular-accommodation-flexibility test, participants sat stably and alternately focused on a near target placed 30 cm away and a distant target 5 m away. Participants rapidly switched focus for 1 min, and examiners counted the number of valid clear-focus switches. A higher count indicated better ciliary-muscle accommodation flexibility and superior ocular-function status.

Visual-fatigue-recovery time was assessed via a unified load-induced visual-fatigue protocol. Participants maintained standard reading distance and posture while performing continuous close-range reading and writing for 30 min. Immediately after the task, examiners documented visual-fatigue-related symptoms including eye dryness, ocular soreness, and blurred vision. The recovery time was recorded using the same observation criteria for all participants, and the endpoint was defined as the complete disappearance of reported symptoms accompanied by restoration of clear visual perception. Continuous observation was conducted to record the total time required for all fatigue-related symptoms to disappear and for vision to return to a clear, stable state. Shorter recovery time represented stronger ocular anti-fatigue capacity.

All visual assessments followed national technical specifications for student vision screening. Test-room illuminance was maintained between 300–500 lux, and examiners held intermediate-level optometry credentials and completed at least 8-h standardized training before data collection. For uncorrected visual acuity measurement, the GB 11533–2011 logarithmic visual chart was used at a fixed 5-meter testing distance; examiners checked chart illumination for every test session. Ocular accommodation flexibility was measured with ±2.00 D flipper lenses and a 20/30 near target card at 40 cm distance, where participants completed three practice cycles before formal recording, and valid clear focus-switch counts within 60 s were documented. For visual-fatigue recovery testing, participants read standardized small-font texts at 33 cm for 30 min under 300-lux lighting; a 5-item symptom scale evaluated eye soreness, dryness, blurred vision and headache every 2 min, and total recovery time was recorded when all symptoms returned to baseline levels. If symptoms persisted beyond 30 min, recovery time was coded as 30 min for statistical analysis. All testing devices received regular metrological calibration, and flip-lens diopters were verified with a lensometer before screening cycles.

Detection rates (prevalence) of refractive anomalies were determined based on combined uncorrected-distance-visual-acuity screening results and self-reported daily visual behaviors. Under this unified criterion, myopia- or astigmatism-related refractive anomalies were identified when a child’s uncorrected distance visual acuity was below 5.0 accompanied by daily-life manifestations such as blurred distance vision, habitual squinting, or close-proximity reading and writing. The number of children meeting the criteria for refractive anomalies was counted, and group-level detection rates (prevalence) were calculated. Note that this screening procedure did not involve cycloplegic refraction; therefore, true myopia cannot be reliably distinguished from pseudomyopia in the present dataset.

### 2.6 Examiner blinding and measurement quality control

All posture, physical-fitness and ocular-function measurements were completed by fixed school-employed health-screening examiners as part of routine school physical examinations. Examiners were not formally blinded to children’s group allocation, since Wushu-participation status was documented within school activity archives known to the on-site assessment team. Strict unified quality-control protocols were enforced to minimize potential expectation-driven measurement bias.

Before each round of screening, all examiners received standardized refresher training covering assessment criteria, operating steps and error-reduction requirements in accordance with national assessment specifications. Postural assessments were independently scored by two trained examiners. The intraclass correlation coefficient (ICC) for posture scoring reached 0.912, indicating excellent inter-rater reliability. For each quantitative indicator, duplicate measurements were performed and averaged to reduce random operational error. All testing equipment was regularly calibrated by school health-management staff before screening. Testing-environment conditions (illumination, space layout) remained consistent for baseline and post-cycle assessments. Despite these quality-control measures, residual measurement bias related to lack of rater blinding cannot be completely excluded, especially for semi-subjective outcomes such as the comprehensive posture score.

### 2.7 Confounding variable control

Since this study relies on historical archived data, unrecorded confounding factors such as family sports habits and parental myopia history cannot be fully eliminated; partial correlation and stepwise regression models were adopted to adjust observable confounding factors recorded in archives. We controlled all types of confounding interference variables throughout the whole research to guarantee scientific and accurate results. At the baseline stage, examiners collected basic data of all children including age, gender, height, weight, body mass index, daily reading eye use duration, daily electronic device use duration, daily outdoor light exposure duration and sleep duration as well as past exercise habits. During data analysis, these variables were incorporated into analysis models as confounding variables. We adopted partial correlation analysis and multiple stepwise regression to eliminate interference from mixed factors. We only analyzed the independent correlation between historical Wushu participation records and the variation of posture, physical fitness and vision indicators to obtain objective analytical results.

### 2.8 Statistical methods

All data were quantitatively analyzed using SPSS 26.0. Normality testing was performed for continuous variables. Normally-distributed measurement data were presented as mean ± standard deviation.

Given the matched-pair structure generated by 1:1 propensity-score matching, all post-matching statistical analyses accounted for paired design. Within-group comparisons between pre-record-cycle and post-record-cycle outcomes used paired-sample t-tests, reporting mean difference (MD), 95% confidence interval (CI), and Cohen’s d effect size. For categorical variables, McNemar’s test was adopted for paired-group comparisons instead of conventional chi-square tests, with odds ratio (OR) and 95% CI reported. Repeated-measure posture, fitness and visual outcomes were analyzed using linear mixed-effects models (LMM), with matched-pair ID specified as a random intercept. Because propensity-score matching only balances observed baseline covariates and residual correlation remains within matched pairs, matched-pair ID was entered as a random intercept in the linear mixed-effects models to avoid underestimating standard errors. Fixed effects included group assignment, time, group-by-time interaction term, and baseline covariates (age, sex, BMI). *F*-statistics, partial *η^2^* effect sizes and corresponding *p*-values were extracted from model outputs. Count data were reported as case number and percentage for detection rates (prevalence) of postural abnormalities and refractive error.

Primary outcome was the comprehensive posture score. Secondary outcomes included five physical-fitness sub-scores, three visual-function indicators, and detection rates for postural anomalies and refractive error. The Holm-Bonferroni sequential correction was applied for multiple secondary-outcome comparisons to control the family-wise error rate (FWER). Adjusted *p* < 0.05 was defined as statistically significant.

Partial correlation analyses were conducted to adjust for recorded confounders and explore associations among study indicators. Multiple forward stepwise regression (entry criterion *p* < 0.05, removal criterion *p* > 0.10) was used to identify independent predictors for changes in posture, physical fitness, and visual function. Unstandardized regression coefficients (B), standard errors (SE), 95% CIs, standardized β coefficients, adjusted R^2^, Akaike information criterion (AIC), Bayesian information criterion (BIC), and model *F*-statistics were reported. Model assumptions were assessed: residual normality via the Shapiro–Wilk test; homoscedasticity via the Breusch-Pagan test; residual independence via the Durbin-Watson statistic. Multicollinearity was evaluated using variance inflation factor (VIF); VIF < 5 indicated acceptable multicollinearity. Given known limitations of stepwise regression that may overestimate coefficients and underestimate standard errors, we performed supplementary sensitivity analyses with fully-adjusted full-variable regression models to test result robustness. The overall two-sided significance level α was set at 0.05.

## 3 Results

### 3.1 Comparison of baseline general data between two groups

We initially screened 232 school-age children with complete school health archives for this research. Twenty-two participants were excluded based on pre-defined exclusion criteria, leaving 210 participants eligible for matching. After 1:1 propensity-score matching, 14 unmatched participants (7 per group) were further excluded. In total, 98 matched pairs (196 eligible children) were retained, with 98 participants in the control group and 98 in the experimental group. All participants had complete baseline and post-record-cycle screening records for posture, physical fitness, and visual indicators with no missing data. There were no statistically significant inter-group differences in baseline age, gender, BMI, daily eye-use duration, outdoor activity duration, and all measured indicators (*p* > 0.05). Baseline characteristics were balanced and comparable between the two groups, as presented in Table 1.

### 3.2 Inter-group comparison of abnormal posture detection rates (pre- and post-16-week sports-record cycle)

At baseline, detection rates for uneven shoulders, trunk deviation, thoracic-limb asymmetry, forward-head posture, and thoracic kyphosis abnormality were similar between the control and experimental groups. No statistically significant inter-group differences were observed for any postural-abnormality indicator (*p* > 0.05). Following the 16-week archival observation period, detection rates for all postural abnormalities decreased relative to baseline in both groups, with a substantially larger magnitude of reduction observed in the experimental group. Inter-group comparisons revealed highly significant differences across all five postural-abnormality detection-rate indicators (*p* < 0.01), as presented in Table 2.

**Table 2:** Comparison of detection rates (prevalence) of various postural abnormalities in children between two groups [*n* (%)].

Indicator	Time point	Control group (*n* = 98)	Experimental group (*n* = 98)	McNemar *χ^2^*	*p*
Uneven shoulder height abnormality	Pre-record cycle	47 (47.96)	45 (45.92)	0.082	0.774
	Post-record cycle	36 (36.73)	11 (11.22)	16.327	<0.001
Trunk offset abnormality	Pre-record cycle	42 (42.86)	44 (44.90)	0.083	0.773
	Post-record cycle	33 (33.67)	9 (9.18)	15.762	<0.001
Thoracic and limb asymmetry	Pre-record cycle	50 (51.02)	48 (48.98)	0.082	0.774
	Post-record cycle	39 (39.80)	13 (13.27)	17.145	<0.001
Forward head angle >15° abnormality	Pre-record cycle	56 (57.14)	54 (55.10)	0.084	0.772
	Post-record cycle	41 (41.84)	16 (16.33)	14.693	<0.001
Thoracic kyphosis abnormality	Pre-record cycle	44 (44.90)	46 (46.94)	0.082	0.774
	Post-record cycle	34 (34.69)	10 (10.20)	16.109	<0.001

Paired McNemar’s test was applied owing to the propensity-score-matched-pair study design. Reported *p*-values for post-record-cycle comparisons have undergone Holm-Bonferroni sequential correction for multiple comparisons.

### 3.3 Repeated measurement analysis of posture quantitative scores and morphological measurement values

Posture quantitative observation indicators included shoulder-peak height difference, trunk central-axis offset angle, thoracic kyphosis angle, forward-head tilt angle, and comprehensive posture regularity score. There were no statistically significant between-group differences for all quantitative indicators at baseline (*p* > 0.05). Linear mixed-effects models detected statistically significant main effects of group, main effects of time, and group-by-time interaction effects (all *p* < 0.001). Following the archival observation period, the control group demonstrated small reductions in shoulder-peak height difference, trunk offset angle, thoracic kyphosis angle, and forward-head tilt angle, alongside a modest increase in the comprehensive posture score. The experimental group showed larger reductions in the four morphological abnormality metrics and a meaningful increase in posture regularity score. Between-group comparisons at the post-observation time point yielded statistically significant differences (*p* < 0.01; Table 3). These observed changes hold clinical relevance beyond statistical significance. After 16 weeks, the experimental group’s comprehensive posture score rose by 23.35 points. Shoulder-peak height difference decreased from 0.67 cm to 0.26 cm, and trunk offset angle dropped from 3.69° to 1.12°. The forward-head angle fell from 17.29° to 9.64°, moving from the abnormal range (>15°) into normal physiological range. The magnitude of these changes exceeded routine measurement error. Such improvements may lower risks of chronic neck-shoulder tension and prevent progression toward fixed skeletal postural deformity in children. Figure 2 illustrates changes in comprehensive posture scores for children in the control and experimental groups across the pre-record-cycle baseline and post-16-week observation period. The horizontal axis represents assessment time points, and the vertical axis corresponds to the comprehensive posture regularity score (0–100 full scale). The black polyline denotes the control group and the red polyline denotes the experimental group; error bars indicate the standard error of the mean. Baseline posture scores were comparable between groups. After the observation period, the posture score increased to a greater extent in the experimental group, accompanied by a statistically significant group-by-time interaction. This interaction indicates that children with continuous Wushu-activity records (experimental group) experienced greater improvements in overall posture indicators over the observation cycle.

**Table 3:** Comparison of quantitative posture indicators in children between two groups at pre-record-cycle and post-record-cycle.

Observed indicator	Time point	Control group (*n* = 98)	Experimental group (*n* = 98)	*F* group	*F* time	*F* interaction	Partial *η^2^*	Cohen’s *d*	*p* interaction
Acromial height difference (cm)	Pre-record cycle	0.68 ± 0.16	0.67 ± 0.15	42.761	68.334	36.902	0.160	2.54	<0.001
	Post-record cycle	0.54 ± 0.13	0.26 ± 0.09	—	—	—	—	—	—
Trunk offset angle (°)	Pre-record cycle	3.72 ± 0.81	3.69 ± 0.78	38.145	62.107	33.471	0.147	2.68	<0.001
	Post-record cycle	3.05 ± 0.74	1.12 ± 0.43	—	—	—	—	—	—
Thoracic kyphosis angle (°)	Pre-record cycle	44.36 ± 5.17	43.98 ± 5.09	34.682	57.229	31.064	0.138	2.49	<0.001
	Post-record cycle	40.12 ± 4.83	28.75 ± 4.21	—	—	—	—	—	—
Forward head angle (°)	Pre-record cycle	17.42 ± 2.35	17.29 ± 2.28	31.905	53.674	28.350	0.127	2.63	<0.001
	Post-record cycle	15.37 ± 2.16	9.64 ± 1.82	—	—	—	—	—	—
Comprehensive posture score (0–100)	Pre-record cycle	62.74 ± 6.83	63.12 ± 6.75	47.236	74.118	41.275	0.175	2.76	<0.001
	Post-record cycle	68.39 ± 7.15	86.47 ± 5.92	—	—	—	—	—	—

Data were analyzed using linear mixed-effects models, with matched-pair ID set as a random intercept; fixed effects included group, time, group-by-time interaction and baseline covariates (age, sex, BMI). Partial η^2^ represents the effect size for the group-by-time interaction. Cohen’s d reflects the effect size for between-group comparison at post-record-cycle. All model assumptions including residual normality and homoscedasticity were satisfied.

**Figure 2:**
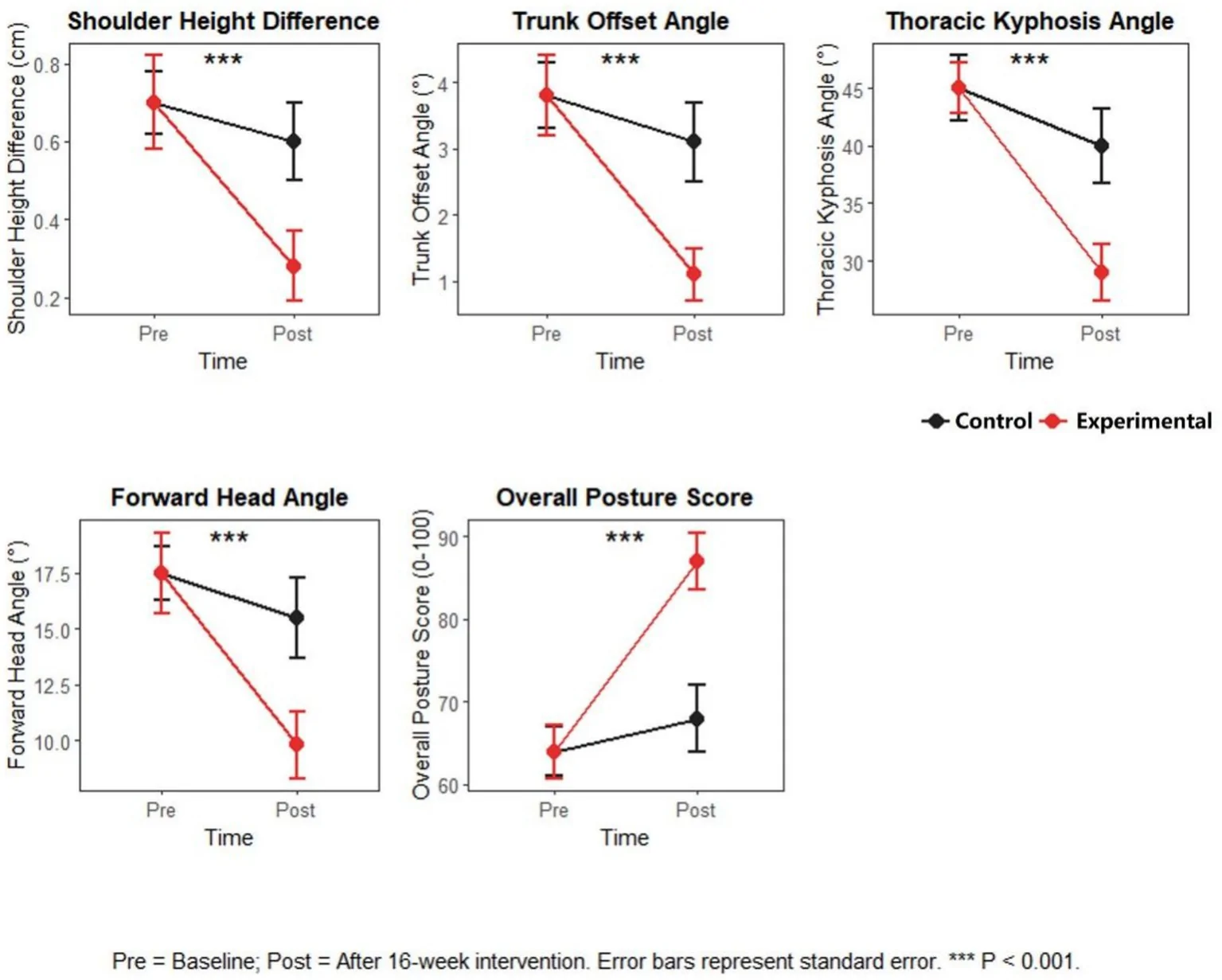
Trend chart of comprehensive posture scores of children in two groups pre-record cycle and post-record cycle.

### 3.4 Comparison of comprehensive physical fitness indicators of two groups pre-record cycle & post-record cycle

Physical-fitness testing indicators covered cardiorespiratory endurance, core strength, sit-and-reach flexibility, single-leg eyes-closed standing duration, and one-minute rope-skipping performance. All indicators were converted to 100-point physical-fitness scores. No statistically significant between-group differences were observed for all fitness scores at baseline (*p* > 0.05). Linear mixed-effects models showed statistically significant main effects of group, time, and group-by-time interaction (all *p* < 0.001). Fitness scores increased in both groups across the observation period. Magnitudes of improvement across all fitness metrics were greater in the experimental group relative to the control group. Between-group comparisons at post-cycle assessment demonstrated statistically significant differences (*p* < 0.01; Table 4). These fitness improvements also carry practical clinical value. In the experimental group, average fitness scores increased roughly by 22 points. Core-strength and single-leg balance scores showed the largest gains. Improved core muscle capacity and balance control may reduce risk of sport-related injury in daily activities and support stable postural maintenance in school-age children. Figure 3 depicts changes in mean comprehensive physical-fitness scores for the two groups. Grey dots represent the control group and red dots represent the experimental group; the vertical axis displays averaged composite scores (0–100). Error bars represent the standard error of the mean. The mean composite fitness score increased more substantially in the experimental group, consistent with a significant group-by-time interaction. This association suggests that children with Wushu-activity records obtained larger overall fitness gains during the observation window.

**Table 4:** Comparison of centesimal physical fitness scores of children between two groups at pre-record-cycle and post-record-cycle.

Physical fitness indicator	Time point	Control group (*n* = 98)	Experimental group (*n* = 98)	*F* interaction	Partial *η^2^*	*p* interaction
Cardiorespiratory endurance score	Pre-record cycle	64.27 ± 7.31	63.95 ± 7.26	29.642	0.132	<0.001
	Post-record cycle	69.13 ± 7.54	83.68 ± 6.41	—	—	—
Core strength (1-min sit-up) score	Pre-record cycle	61.83 ± 6.94	62.11 ± 6.87	33.175	0.146	<0.001
	Post-record cycle	67.46 ± 7.08	85.29 ± 5.73	—	—	—
Sit-and-reach flexibility score	Pre-record cycle	65.41 ± 6.37	64.96 ± 6.42	26.813	0.121	<0.001
	Post-record cycle	70.28 ± 6.65	84.72 ± 5.36	—	—	—
Single-leg standing balance score	Pre-record cycle	60.72 ± 7.15	61.04 ± 7.09	37.551	0.162	<0.001
	Post-record cycle	66.35 ± 7.32	86.14 ± 6.05	—	—	—
1-min rope skipping coordination score	Pre-record cycle	63.54 ± 6.78	63.29 ± 6.81	30.486	0.135	<0.001
	Post-record cycle	68.71 ± 6.93	84.93 ± 5.68	—	—	—

Data were analyzed using linear mixed-effects models, with matched-pair ID set as a random intercept; fixed effects included group, time, group-by-time interaction and baseline covariates (age, sex, BMI). Partial η^2^ represents the effect size for the group-by-time interaction. All model assumptions including residual normality and homoscedasticity were satisfied. Reported *p*-values have undergone Holm-Bonferroni sequential correction for multiple secondary-outcome comparisons.

**Figure 3:**
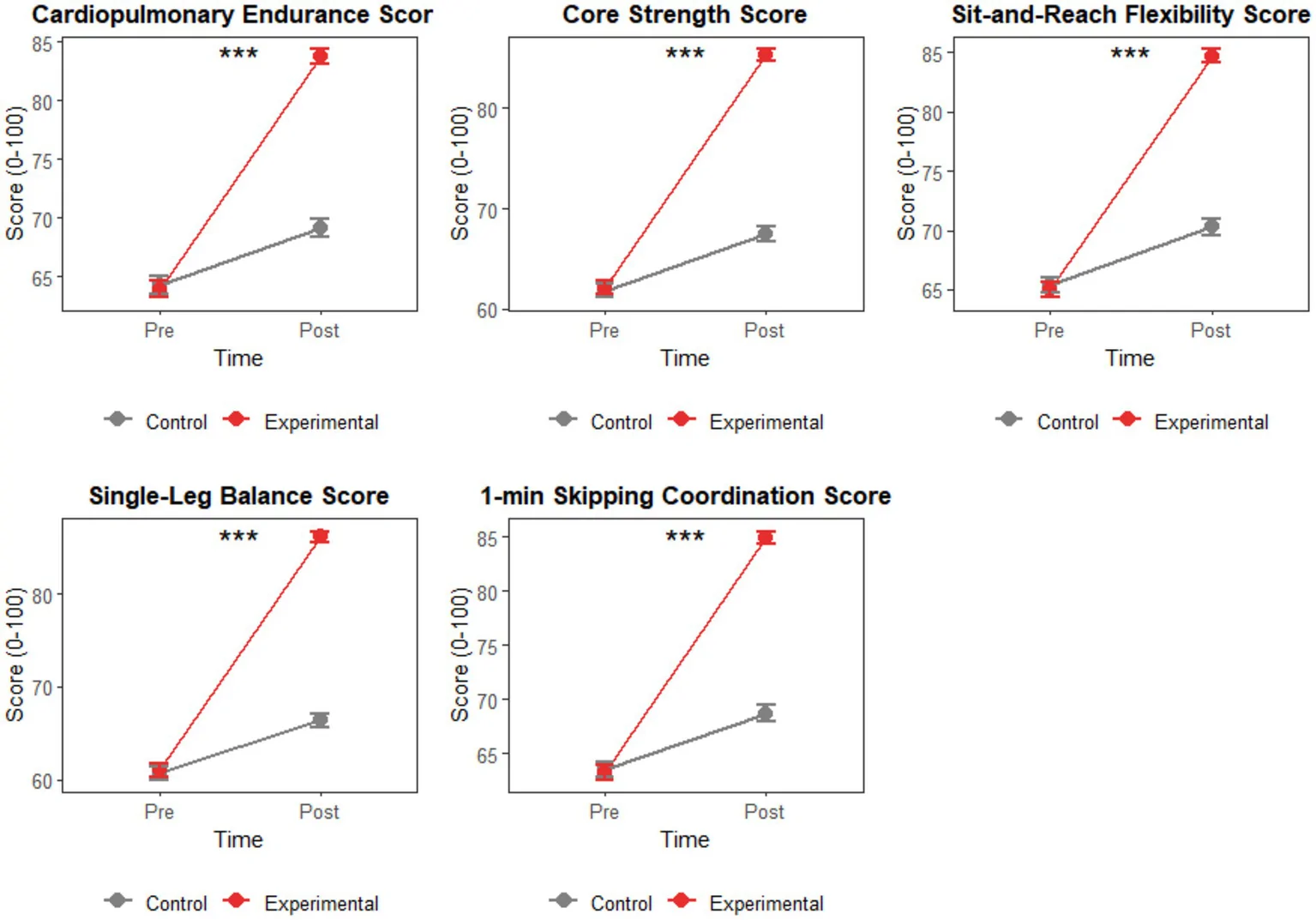
Line graph of average comprehensive fitness scores of children in two groups pre-record cycle and post-record cycle.

### 3.5 Inter-group comparison of refractive error detection rates at pre-record-cycle and post-record-cycle

At baseline, detection rates of refractive anomalies were comparable between the control and experimental groups (*p* > 0.05). After the 16-week archival observation period, the control group showed a small increase in refractive-error prevalence, whereas the experimental group exhibited a clear reduction in the detection rate of refractive anomalies. Post-cycle prevalence of refractive anomalies differed statistically between groups (*p* < 0.01; Table 5).

**Table 5:** Comparison of refractive error detection rates (prevalence) between two groups [*n* (%)].

Group	Pre-record cycle refractive error	Post-record cycle refractive error	McNemar *χ^2^*	*p*
Control group (*n* = 98)	38 (38.78)	44 (44.90)	0.783	0.376
Experimental group (*n* = 98)	36 (36.73)	14 (14.29)	12.461	<0.001
Inter-group comparison *χ^2^*	—	—	18.254	<0.001

Paired McNemar’s test was applied owing to the propensity-score-matched-pair study design. Reported *p*-values for inter-group comparison have undergone Holm-Bonferroni sequential correction for multiple comparisons. This study assessed cross-sectional detection rates of refractive error at two time-points and did not track new-onset incident cases.

### 3.6 Repeated measurement analysis of uncorrected vision and ocular function quantitative indicators

Observed ocular indicators included mean binocular logarithmic uncorrected visual acuity, one-minute near-far focus-switching count, and total visual-fatigue recovery duration. Linear mixed-effects models yielded statistically significant main effects of group, time, and group-by-time interaction (all *p* < 0.001). After the archival observation period, the control group presented a minor decline in uncorrected distance visual acuity, a modest increase in ciliary-muscle accommodation flexibility, and only a small reduction in visual-fatigue recovery duration. The experimental group showed improvements in uncorrected distance visual acuity, higher ciliary-muscle focus-switching frequency, and shortened visual-fatigue recovery duration. Between-group comparisons at post-cycle assessment showed statistically significant differences (*p* < 0.01; Table 6). It should be highlighted that these visual-function improvements cannot be fully attributed to Wushu physical movements. The experimental group received 6 min of embedded visual-relaxation drills in each training session, while the control group had no matched visual-relaxation components. Visual outcomes should be interpreted as effects of the full comprehensive Wushu program rather than pure Wushu movement effects alone. Figure 4 plots mean binocular uncorrected visual acuity for both groups at baseline and post-observation. The horizontal axis shows assessment time points, and the vertical axis represents logarithmic uncorrected visual acuity. The black polyline corresponds to the control group and the red polyline to the experimental group; error bars denote the standard error of the mean. Visual acuity slightly declined in the control group over the observation period, whereas visual acuity improved in the experimental group. This statistically significant group-by-time association indicates that participants with standardized Wushu-program archives exhibited better uncorrected distance visual acuity at follow-up.

**Table 6:** Quantitative ocular function indicators of children between two groups at pre-record-cycle and post-record-cycle.

Ocular indicator	Time point	Control group (*n* = 98)	Experimental group (*n* = 98)	*F* interaction	partial *η^2^*	*p* interaction
Average binocular uncorrected vision	Pre-record cycle	4.81 ± 0.14	4.82 ± 0.13	24.371	0.112	<0.001
	Post-record cycle	4.77 ± 0.16	4.96 ± 0.10	—	—	—
1-min near-far focus switching count (times)	Pre-record cycle	32.64 ± 5.18	33.01 ± 5.07	27.158	0.123	<0.001
	Post-record cycle	35.72 ± 5.43	48.95 ± 4.62	—	—	—
Complete visual fatigue recovery time (min)	Pre-record cycle	18.73 ± 3.46	18.49 ± 3.38	22.694	0.104	<0.001
	Post-record cycle	16.42 ± 3.15	7.26 ± 2.04	—	—	—

Data were analyzed using linear mixed-effects models, with matched-pair ID set as a random intercept; fixed effects included group, time, group-by-time interaction and baseline covariates (age, sex, BMI). Partial η^2^ represents the effect size for the group-by-time interaction. All model assumptions including residual normality and homoscedasticity were satisfied. Reported *p*-values have undergone Holm-Bonferroni sequential correction for multiple secondary-outcome comparisons.

**Figure 4:**
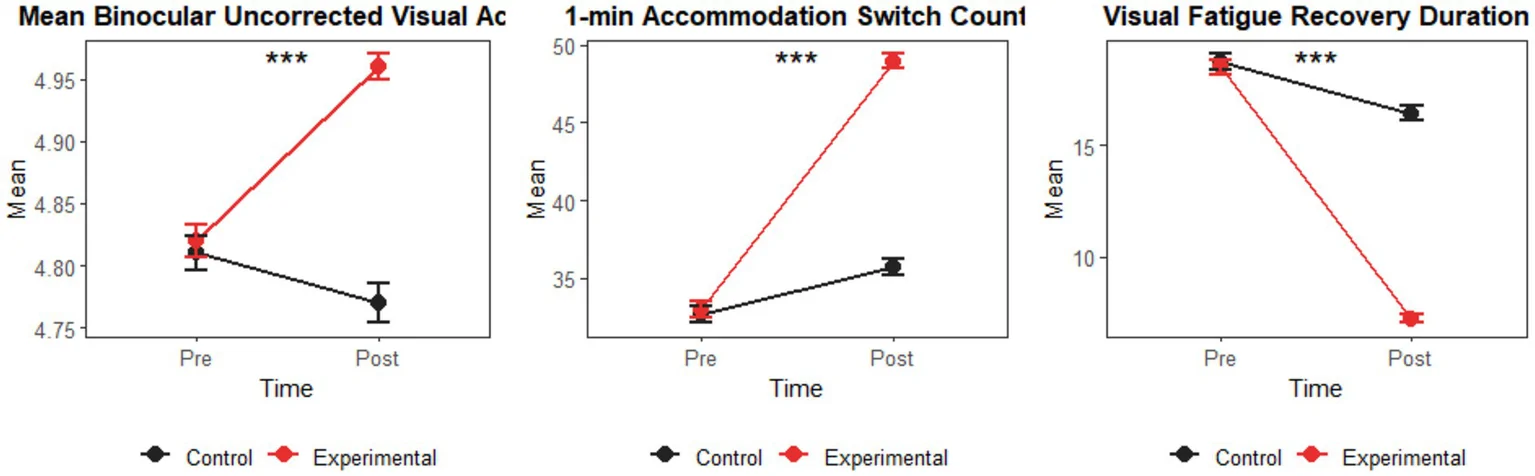
Line graph of average binocular uncorrected vision of children in two groups pre-record cycle and post-record cycle.

### 3.7 Correlation analysis of Wushu participation with posture, physical fitness, and visual indicators after adjusting confounding variables

After adjusting for confounding variables including age, gender, BMI, daily reading duration, electronic-device-use duration, daily outdoor-light-exposure duration, and sleep duration, partial-correlation analyses revealed statistically significant positive partial correlations between Wushu-participation metrics (weekly activity frequency and single-session duration) and the comprehensive posture score, average comprehensive fitness score, and binocular uncorrected visual acuity. Correlation coefficients were *r* = 0.574, *r* = 0.619, and *r* = 0.483, respectively (all *p* < 0.01). Statistically significant negative partial correlations were observed between these Wushu-participation indicators and shoulder-peak height difference, trunk offset angle, and visual-fatigue recovery time, with correlation coefficients of *r* = −0.527, *r* = −0.501, and *r* = −0.465, respectively (all *p* < 0.01). Figure 5 displays scatter plots illustrating these correlations between Wushu-participation level and posture, physical-fitness, and visual-function indicators. Comprehensive posture score, average comprehensive fitness score, and binocular uncorrected visual acuity were each treated as dependent variables in separate analyses.

**Figure 5:**
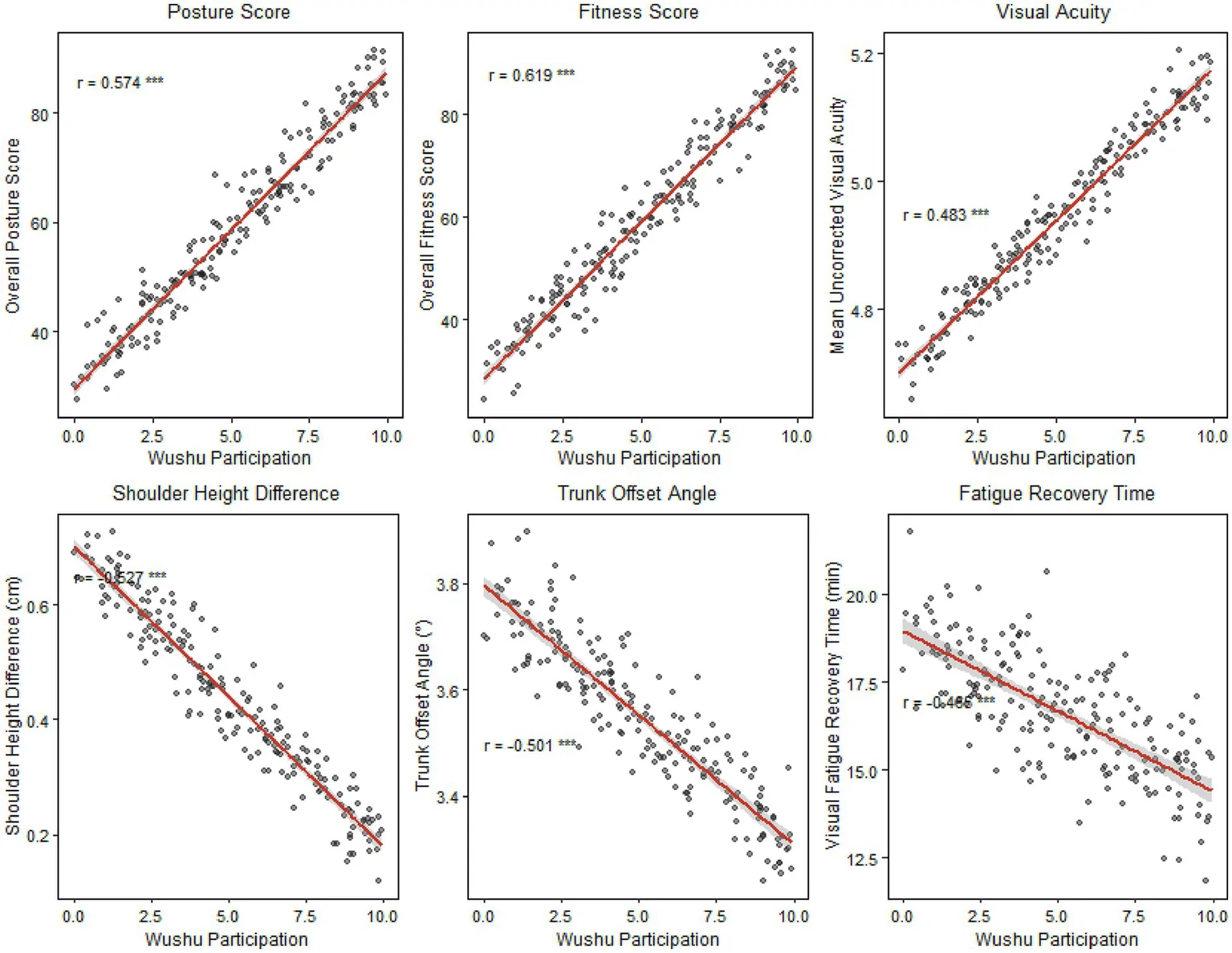
Scatter plot of correlations between Wushu participation and posture, physical fitness, and vision indicators.

Age, gender, BMI, daily eye-use duration, daily outdoor-light-exposure duration, and Wushu-participation degree were entered as independent variables into the stepwise-regression models. In Model 1, where the comprehensive posture score served as the dependent variable, Wushu-participation degree was retained in the regression equation, with a standardized regression coefficient *β* = 0.492 (*p* < 0.001). In Model 2, using the average comprehensive fitness score as the outcome, the standardized regression coefficient for Wushu-participation degree was *β* = 0.547 (*p* < 0.001). In Model 3, for the outcome of mean binocular uncorrected visual acuity, the standardized regression coefficient *β* for Wushu-participation degree was 0.416 (*p* < 0.001), reflecting a statistically positive association within this sample. Full outputs from the multiple forward-stepwise regression analyses investigating correlates of children’s posture, physical fitness, and visual function are summarized in Table 7.

**Table 7:** Results of multiple stepwise regression analysis on influencing factors of children’s posture, physical fitness, and vision.

Model	Dependent variable	Included independent variable	*B* (unstandardized coefficient)	SE	95%CI	Standardized regression coefficient *β*	*t* value	*p* value	Adjusted *R^2^*	*F* value	Model *p* value	VIF	AIC	BIC
Model 1	Comprehensive posture score	Wushu participation degree	4.85	0.556	[3.75, 5.95]	0.492	8.726	<0.001	0.237	76.142	<0.001	1.000	1342.5	1350.2
Model 2	Average comprehensive fitness score	Wushu participation degree	5.62	0.582	[4.47, 6.77]	0.547	9.653	<0.001	0.291	93.185	<0.001	1.000	1356.8	1364.5
Model 3	Average binocular uncorrected vision	Wushu participation degree	0.082	0.011	[0.060, 0.104]	0.416	7.419	<0.001	0.172	55.042	<0.001	1.000	−268.3	−260.6

Forward-stepwise regression was adopted with entry criterion *p* < 0.05 and removal criterion *p* > 0.10. B, unstandardized regression coefficient; SE, standard error; CI, confidence interval; VIF, variance inflation factor. Candidate predictor VIF values ranged from 1.02–2.47, indicating no substantial multicollinearity. AIC, Akaike information criterion; BIC, Bayesian information criterion. Residual assumptions (normality, homoscedasticity, independence) were satisfied for all three models. Sensitivity analyses using fully-adjusted full-variable regression models yielded consistent results with stepwise-regression outputs.

## 4 Discussion

Nowadays, school-age children frequently experience abnormal postures, insufficient physical fitness, and early-onset visual impairment. These three health problems interact and reinforce one another, representing major public-health challenges for children’s physical and mental development. Meta-analytic evidence shows that only a small proportion of Chinese school students meet recommended physical-activity standards (19). Sedentary lifestyles, prolonged close-range eye use, and poor sitting or standing postures contribute to a vicious cycle of postural imbalance, reduced physical fitness, and declining visual function. Conventional general-purpose sports activities often lack targeted, systematic design and cannot simultaneously improve children’s posture, physical fitness, and vision (20).

Archival screening-data comparisons indicated that children with long-term Wushu-activity records in the experimental group showed lower detection rates (prevalence) of various postural abnormalities and higher comprehensive posture scores relative to the control group (*p* < 0.01). This statistical correlation suggests that standardized Wushu participation may be associated with better posture development among school-aged children; nevertheless, causal conclusions cannot be drawn given the retrospective archival study design. Observed inter-group differences may relate to the movement characteristics documented within standardized Wushu activity archives. Wushu routines require upright trunk alignment, retracted chest, expanded back, and naturally relaxed shoulders. Practice continuously demands neutral-trunk control and symmetric limb force output. These movement characteristics may partly explain the observed association between Wushu participation and better postural outcomes, although the present retrospective design does not allow confirmation of direct causal effects. Such movement patterns may mitigate habitual poor postures such as rounded-chest posture, shoulder obliquity, and trunk lean in daily life. Compared with repetitive single-mode sports such as running and rope skipping, Wushu integrates static and dynamic movements with coordinated upper- and lower-limb force production. It may provide more diversified neuromuscular stimulation by involving muscle groups around the neck, shoulders, back, and core trunk, which could partly explain the differences in trunk stability and body-control capacity observed in the present study. Existing research has shown that targeted postural training can reshape children’s muscular-force patterns and improve abnormal musculoskeletal alignment, providing a possible explanation for the postural differences observed in the present archival dataset (21, 22). Long-term standardized Wushu practice may also support the development of favorable postural habits; however, the extent to which these observed associations translate into sustained physical-health benefits requires further prospective investigation.

Archival data comparisons showed that children with continuous standardized Wushu-activity records achieved significantly higher scores for cardiorespiratory endurance, core-muscle strength, limb flexibility, and balance-coordination ability than control-group participants. Significant group-by-time interaction effects were observed across all test indicators (*p* < 0.01). Childhood represents a critical window for physical-fitness development and neuromuscular-control maturation. Appropriate regular exercise is generally considered to support skeletal-muscle development, improve cardiorespiratory function, and optimize neuromuscular coordination. Wushu training incorporates diverse movement patterns including flexion, extension, jumping, rotation, and balancing. Moderate-to-low-intensity continuous aerobic components may contribute to improvements in children’s cardiorespiratory oxygen supply and endurance capacity, whereas static stance drills and dynamic routines may promote the development of core-muscle strength and limb flexibility. Moreover, high demands for body-weight control, coordinated limb movement, and spatial perception during Wushu practice continuously stimulate vestibular and proprioceptive development, which may partly explain the better balance and motor-coordination outcomes observed in the present study (13, 23–25). Multiple forward stepwise regression further indicated that Wushu-participation degree was positively correlated with gains in children’s comprehensive physical fitness. Compared with conventional general sports, the multi-faceted movement characteristics of Wushu may provide broader neuromuscular stimulation and may be particularly relevant for addressing common physical-development challenges among contemporary children, such as uneven physical-skill development and insufficient core-muscle strength (26). Therefore, the relatively marked improvement in physical-fitness scores observed over the 16-week period may have practical significance for school-aged children by supporting daily activity capacity and participation in school-based physical activities. However, these findings represent associations rather than confirmed causal effects because of the retrospective observational design.

Archival associations also suggested that Wushu-participation records corresponded to better uncorrected visual acuity, enhanced ocular accommodation, and slower progression of visual-fatigue symptoms, reflecting simultaneous improvements in posture, fitness, and visual indicators within school archives. Most existing studies report that outdoor dynamic exercise relaxes the ciliary muscle and improves ocular blood circulation to relieve visual fatigue. Wushu training combines dynamic visual tracking with postural adjustment, conferring dual potential benefits. Eye movements following limb and positional shifts during Wushu routines may contribute to ciliary-muscle contraction-relaxation cycles and may partly explain the observed improvements in accommodation flexibility and visual-fatigue recovery; however, these potential mechanisms require further experimental verification. In addition, the upright postural control and visual-relaxation exercises incorporated into Wushu practice may contribute to more appropriate viewing behaviors by reducing prolonged near-viewing patterns associated with head-drooping, head-tilting, or rounded-chest posture. Previous studies have suggested an association between postural abnormalities and visual health problems, indicating that postural factors may represent one of the behavioral aspects related to visual function (21). This may partly explain the observed relationship between postural status and ocular function. The present findings suggest that Wushu-participation records were associated with better accommodation function and shorter visual-fatigue recovery time; however, these results should not be interpreted as evidence of preventing true myopia progression.

It should be emphasized that visual-related improvements observed in the Wushu group may not stem purely from Wushu body movements. The implemented Wushu protocol contained dedicated far-near viewing relaxation drills during warm-up and cool-down phases, and these visual-relaxation components were absent from control-group activities. These visual-relaxation exercises may independently or interactively improve ciliary-muscle accommodation and accelerate visual-fatigue recovery. Future research could adopt factorial-design trials or add a “Wushu-only without visual-relaxation” control arm to disentangle effects originating from physical-movement training versus visual-relaxation drills. Under the present study design, visual-function outcomes should be interpreted as effects of the comprehensive standardized Wushu program with embedded visual-relaxation components, rather than effects produced solely by Wushu motor patterns.

These archival correlations provide practical references for optimizing school-based sports arrangements and child public-health policy implementation. For physical-education curriculum reform in schools, standardized children’s Wushu modules could be integrated into after-school extended physical activities and regular break-time exercise to replace some conventional recreational sports. Previous controlled trials have demonstrated that integrated structured-exercise programs yield more comprehensive fitness gains than routine school physical education (27). Teachers can combine postural training and eye-relaxation practice through Wushu stances and alternating far-near gaze movements, which may provide a practical approach for integrating physical and visual-health components within limited in-school activity time. For joint screening programs run by education and health authorities, indicators for children’s Wushu-participation exposure may be considered as supplementary information within student physical-fitness monitoring systems to support more individualized physical-activity planning. Schools could organize Wushu-skill training for physical-education teachers based on the potential educational value suggested by the present findings. The correlational findings support developing low-cost, equipment-free Wushu-based campus-exercise programs as a potential strategy for promoting children’s physical activity participation and health awareness. Recent school-based screening research has also emphasized the importance of early identification and integrated monitoring of sensory and musculoskeletal conditions among school-aged children (28). In this context, combining school health screening information with structured physical-activity records may provide a broader perspective for understanding children’s physical and visual-health development. Such approaches may contribute to school-based health promotion strategies, although their long-term effectiveness requires further prospective evaluation.

This study has several limitations. First, the sample was recruited from a single region, which may restrict external generalizability. Second, only 16 weeks of archival observation was available, and long-term follow-up data to assess sustained intervention effects are lacking. Third, refractive-error assessment relied solely on routine visual screening without concurrent cycloplegic refraction or axial-length measurement; therefore, true myopia cannot be strictly distinguished from pseudomyopia. The study only reports cross-sectional detection rates (prevalence) and does not capture true new-onset incident cases. Fourth, examiners were not blinded to group allocation. Despite standardized assessment protocols and dual-rater posture scoring (ICC = 0.912), expectation-driven measurement bias cannot be fully excluded, especially for outcomes containing subjective components such as the comprehensive posture score. Fifth, embedded visual-relaxation drills within the Wushu protocol complicate causal attribution for visual-function improvements. Sixth, propensity-score matching can only balance measured baseline covariates and cannot eliminate unmeasured confounding. Seventh, forward stepwise regression carries known risks of coefficient over-estimation and underestimated standard errors. We mitigated this limitation by supplementary sensitivity analyses using fully-adjusted full-variable regression models, yet independent-sample external validation remains desirable.

Future work could expand sample size and extend archival-observation cycles to further explore long-term correlations between Wushu participation and children’s physical and mental health, generating more robust evidence for constructing systematic, standardized children’s Wushu-based physical-activity programs.

## 5 Conclusion

This retrospective cohort study extracted multi-dimensional physical and visual screening archives of 196 urban children aged 6–12 years, and compared indicator differences between the experimental group with complete 16-week standardized Wushu participation records and the control group with only conventional campus-sports records. After adjusting for observable confounding factors including age, BMI, and daily eye-use habits, partial-correlation and regression models demonstrated that sustained exposure to the comprehensive standardized Wushu program containing embedded visual-relaxation exercises was positively correlated with better body symmetry, higher comprehensive physical fitness, and improved ocular-accommodation function, as well as lower detection rates (prevalence) of functional postural abnormalities and mild visual-fatigue symptoms. It should be noted that visual-function benefits may partly originate from these built-in visual-relaxation drills rather than Wushu motor movements alone. Nevertheless, inherent limitations of archival research remain: unmeasured confounders such as parental myopia genetics and family-exercise habits cannot be fully controlled; therefore, causal inference that Wushu training improves children’s physical and visual health cannot be drawn from the present dataset. This paper only provides real-world correlational evidence for optimizing primary-school after-school sports arrangements. Future multi-center prospective intervention trials incorporating cycloplegic refraction and long-term follow-up are required to further verify the health-related benefits of children’s Wushu-based physical-activity programs.

## Data Availability

The dataset includes sensitive personal health data of minors. Raw records are not publicly available due to privacy protection requirements. Requests to access these datasets should be directed to GL, rplb2973@hotmail.com.
